# From waste to wealth: Repurposing slaughterhouse waste for xenotransplantation

**DOI:** 10.3389/fbioe.2023.1091554

**Published:** 2023-02-03

**Authors:** Raheema L. Khan, Ali A. Khraibi, Ludovic F. Dumée, Peter R. Corridon

**Affiliations:** ^1^ Department of Immunology and Physiology, College of Medicine and Health Sciences, Khalifa University of Science and Technology, Abu Dhabi, United Arab Emirates; ^2^ Center for Biotechnology, Khalifa University of Science and Technology, Abu Dhabi, United Arab Emirates; ^3^ Department of Chemical Engineering, College of Engineering, Khalifa University of Science and Technology, Abu Dhabi, United Arab Emirates; ^4^ Research and Innovation Center on CO_2_ and Hydrogen (RICH), Khalifa University of Science and Technology, Abu Dhabi, United Arab Emirates; ^5^ Healthcare Engineering Innovation Center, Khalifa University of Science and Technology, Abu Dhabi, United Arab Emirates

**Keywords:** slaughterhouse waste, wastewater, repurposing, bioartificial tissues and organs, decellularization, urine-derived stem cells, fecal-derived stem cells

## Abstract

Slaughterhouses produce large quantities of biological waste, and most of these materials are underutilized. In many published reports, the possibility of repurposing this form of waste to create biomaterials, fertilizers, biogas, and feeds has been discussed. However, the employment of particular offal wastes in xenotransplantation has yet to be extensively uncovered. Overall, viable transplantable tissues and organs are scarce, and developing bioartificial components using such discarded materials may help increase their supply. This perspective manuscript explores the viability and sustainability of readily available and easily sourced slaughterhouse waste, such as blood vessels, eyes, kidneys, and tracheas, as starting materials in xenotransplantation derived from decellularization technologies. The manuscript also examines the innovative use of animal stem cells derived from the excreta to create a bioartificial tissue/organ platform that can be translated to humans. Institutional and governmental regulatory approaches will also be outlined to support this endeavor.

## 1 Introduction

Slaughterhouses generate billions of tons of biological waste annually (Montford and Wotherspoon, 2021). The overwhelming majority of this waste is derived from animals that produce meat for human consumption, while the remainder is mainly derived from knackeries and is unfit for consumption. Livestock most commonly slaughtered for food include avian, aquatic, monogastric, and ruminant animals. Some underutilized remnants, including offals, bones, tendons, and blood, are edible and enjoyed as delicacies in certain countries. Nevertheless, approximately 60% of these remnants become waste that must be discarded or recycled, often at considerable cost (Franke-Whittle and Insam, 2013).

**FIGURE 1:**
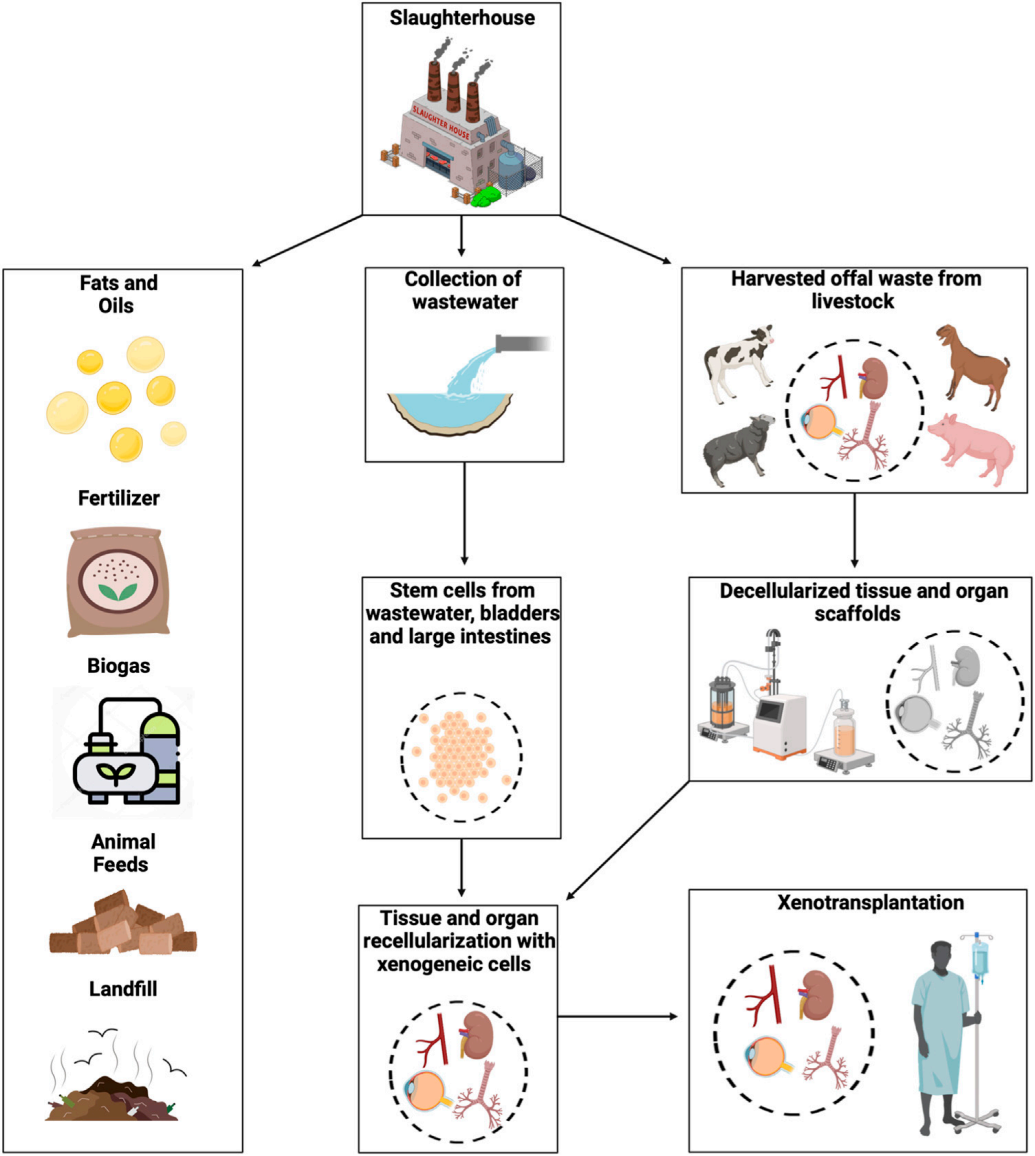
An overview of utilizing slaughterhouse waste for xenotransplantation. The image shows a pathway to harvest biological waste and wastewater from slaughterhouses that can be transported to research facilities. The schematic also outlines ways such biological waste has traditionally been repurposed as fats and oils, fertilizers, biogas, and animal feed. Besides, the waste that is not generally recycled is sent to landfills or can be used to develop bioartificial tissues and organs. These discarded materials can then be processed into acellular templates, and stem cells may be extracted from the excreta to support the development of replacement tissues and organs and bioeconomic practices.

The most common method used to repurpose slaughterhouse waste involves their conversion by rendering plants into industrial byproducts such as fats and oils, in the form of lard and tallow (Chakraborty et al., 2014), fertilizers derived from organic compost (Darch et al., 2019), biogas through methane production (Ware and Power, 2016), and animal feed as meat powder (Ragályi and Kádár, 2012), as shown in Figure 1. Yet, given stricter regulations on the processing of carcasses, rendering has become costly (Franke-Whittle and Insam, 2013). As a result, alternative methods of managing slaughterhouse waste have been sought to offset production costs and increase their potential utility. To this end, some other uses of discarded byproducts have been investigated, including biosubstrates and biomaterials. In turn, this relatively unexplored use of slaughterhouse waste as starting materials has devised a new field focused on generating bioartificial tissues and organs from these remnants. This approach can create the basis for industrial-scale efforts that simultaneously drive circular bioeconomic sustainability and healthcare practices by valued-added repurposing of local slaughterhouse waste in an eco-friendly manner (Pantic et al., 2023a; Wang et al., 2023).

While transplantation is the most effective treatment for end-stage tissue damage and organ failure, an imbalance in supply and demand for human structures remains a hindrance to clinical transplantation (Lu et al., 2020). Employing slaughterhouse waste as a starting material can serve to not only mitigate the shortage of transplantable tissue but also provide an alternative method of treating slaughterhouse waste, as outlined in Figure 1. Several factors must be considered when implementing such waste as a starting material in xenotransplantation. These factors include the utilization of various animal-derived offals in creating bioartificial components that face hindrances such as mimicking native tissue structures, biocompatibility, procuring discarded materials, ethical considerations, and both policies and regulations. Replacing diseased or damaged tissues with tissue-engineered structures that closely resemble the native tissue’s functionality, biology, mechanics, and cellular and extracellular matrix (ECM) compositions is the ultimate goal of tissue regeneration (Griffith and Naughton, 2002).

Maintaining the intrinsic architecture of tissue-engineered structures is particularly important since this characteristic affects function, biomechanics, and cellular behavior. It is also necessary to ensure graft integrity and biocompatibility. Specifically, these materials need to withstand the transplantation environment, as graft failure is a significant concern and is related to the challenges in maintaining viable vascular tracks within these structures. Furthermore, efforts must be made to suppress harmful or immunological responses that lead to hyperacute rejection (Remes et al., 1992; Groth, 2007). Immunosuppressants, transgenic animal models, and cloning procedures are being investigated to minimize the danger of organ rejection.

In this perspective, the viability and sustainability of employing decellularization strategies on slaughterhouse waste as starting materials in xenotransplantation, including blood vessels, eyes, kidneys, and tracheas, will be investigated. At the same time, the routes whereby urine and fecal-derived stem cells may be used to aid the innovative creation of bioartificial structures will also be explored. Furthermore, the regulatory framework necessary to make this possible will also be examined.

## 2 Viability and sustainability of xenografts derived from slaughterhouse waste

It is widely established that various criteria are crucial for the development of xenograft-based tissue-engineering platforms. Specifically, these structures need to support intrinsic physiological functions, biocompatibility, and somatic growth akin to native tissues and organs (Wang et al., 2022a; Shakeel and Corridon, 2023a). Arguably, some of the most challenging parts of creating viable tissue-engineered xenografts include ensuring that they accurately mimic native tissues and limit immunogenicity (Fishman, 2018; Platt et al., 2018; Capella-Monsonís and Zeugolis, 2021; Wang et al., 2022a; Liu et al., 2022). These features are essential in providing a possible solution to the scarcity of transplantable tissues and organs.

In preclinical and clinical investigations, ECM scaffolds are frequently used for reconstructive and regenerative applications (Brown and Badylak, 2014; Aamodt and Grainger, 2016; Nowocin et al., 2016; Xu et al., 2017; Jiang et al., 2021; Anderson et al., 2022; Barbon et al., 2022; Barbulescu et al., 2022; Wang et al., 2022b; Yesantharao and Nguyen, 2022; Zhang et al., 2022; Shakeel and Corridon, 2023a). Naturally occurring ECM-based biopolymers derived from harvested human (Kumar Kuna et al., 2018) and animal (Kumar Kuna et al., 2018) organs and tissues, as well as agarose (Salati et al., 2020), alginate (Ahmad Raus et al., 2021), chitosan (Sultankulov et al., 2019), and cellulose (Silva et al., 2022), can stimulate angiogenesis, growth, and differentiation of repopulating cells. These substrates can also support ECM components’ deposition, organization, and maturation. Such features can, in turn, prevent cell-mediated site contraction and induce tissue remodeling to support the viability and sustainability of various xenografts derived from vascular, ocular, renal, and respiratory lineages (Nowocin et al., 2016; Wang et al., 2022b).

### 2.1 Blood vessels

Vascular replacement therapy (VRT) helps millions of patients overcome life-threatening diseases and traumatic injuries annually. Existing approaches rely on extracting vascular segments from various locations in the thorax and bodily extremities. Unfortunately, such practices can cause severe complications at the donor site. Typical donor-site comorbidities include infections, dehiscence (wound separation), hematomas, restricted movement, chronic pain, and loss of function (Ferraro et al., 2022). The aesthetic outcomes of these surgical procedures often have severe social and psychological impacts on the patients (Honigman et al., 2004). Thus, VRT comorbidities can negatively impact a patient’s quality of life and introduce significant financial burdens if further corrective and cosmetic procedures are required.

These issues have prompted the need for alternative solutions. As a result, efforts have been made to generate grafts capable of limiting the detrimental induction of thrombosis immune responses and compliance mismatches post-transplantation (Khoffi et al., 2014) while maintaining growth capacity, morphology, and patency (Hibino et al., 2015) using prosthetic grafts. Xenotransplantation is also a promising alternative to providing tissues that can bridge the gap between supply and demand and reduce or eliminate the reliance on autologous grafting (Lu et al., 2020). Vascular grafts should ideally integrate structurally and functionally into the recipient, withstand and adapt to physiological hemodynamic responses relative to the site within the cardiovascular system, and be readily available whenever needed (Wilhelmi et al., 2014).

Historically, it has been challenging to achieve these goals. Nevertheless, various classical tissue engineering paradigms have been employed to help generate transplantable vascular segments. Such traditional approaches rely on the isolation, differentiation, and expansion of tissue-specific and, if possible, cells derived *via* autologous transplantation on suitable matrix scaffolds. The resulting combinations of living cells with natural, synthetic, or bioartificial cells support in attempts to create vessel conduits structurally, mechanically, and functionally equal to tissue after *in vitro* development (Caddeo et al., 2017). Alternative methods of generating blood vessel substitutes that are not based on traditional tissue engineering paradigms have also been developed. These approaches have been classified as biology- or material-driven (Wilhelmi et al., 2014). Biology-driven techniques harness the innate cellular capacities to generate ECM components without the support of exogenous scaffolds and have been referred to as scaffold-free approaches. In comparison, material-driven techniques rely on the host reaction for *in situ* tissue engineering after the implantation of cell-free scaffolds.

Within recent years, several research studies have used decellularization-based strategies, combined with tissue engineering principles, to investigate the potential of generating vascular xenografts from slaughterhouses to treat various cardiovascular issues. Either way, generating bioartificial blood vessels from preformed native vessels, which can be obtained from slaughterhouses, provides inherent tissue-specific geometry and biomechanical stability. Furthermore, the potentially limitless supply and comparable size of bovine, porcine, ovine, and hircine vessels obtained from abattoir livestock can produce a scalable repertoire of replacement segments to match human recipients.

For instance, Ran et al. (2019) combined decellularization and electrospinning technologies to generate small-diameter vascular grafts, with inner diameters less than 6 mm, composed of a poly (l-lactide-co-carpro- lactone)/gelatin outer layer and an acellular porcine inner layer that was reinforced with heparin to enhance biomechanical stability and reduce the potential for coagulation. Similarly, pig-to-rat xeno-transplantations performed by Funamoto et al. (2010) highlighted how decellularized blood vessels endure arterial blood pressure and maintain patency within the post-transplantation environment. Furthermore, within this environment, cellular infiltration into the vascular segment was observed four weeks after implantation.

Likewise, efforts by Seiffert et al. (2021) to support the development of autologous tissue-engineered vascular grafting illustrate how vascular xenografts derived from decellularized bovine carotid arteries recellularized with human endothelial colony forming cells may one day serve as a platform to develop small-diameter vessels. Simsa et al. (2019) demonstrated methods to develop large-caliber (inner diameter vessels greater than 6 mm) vessels using the porcine vena cava. Results from this study outline the relatively straightforward manner in which acellular grafts can be generated using various vascular segments.

**FIGURE 2:**
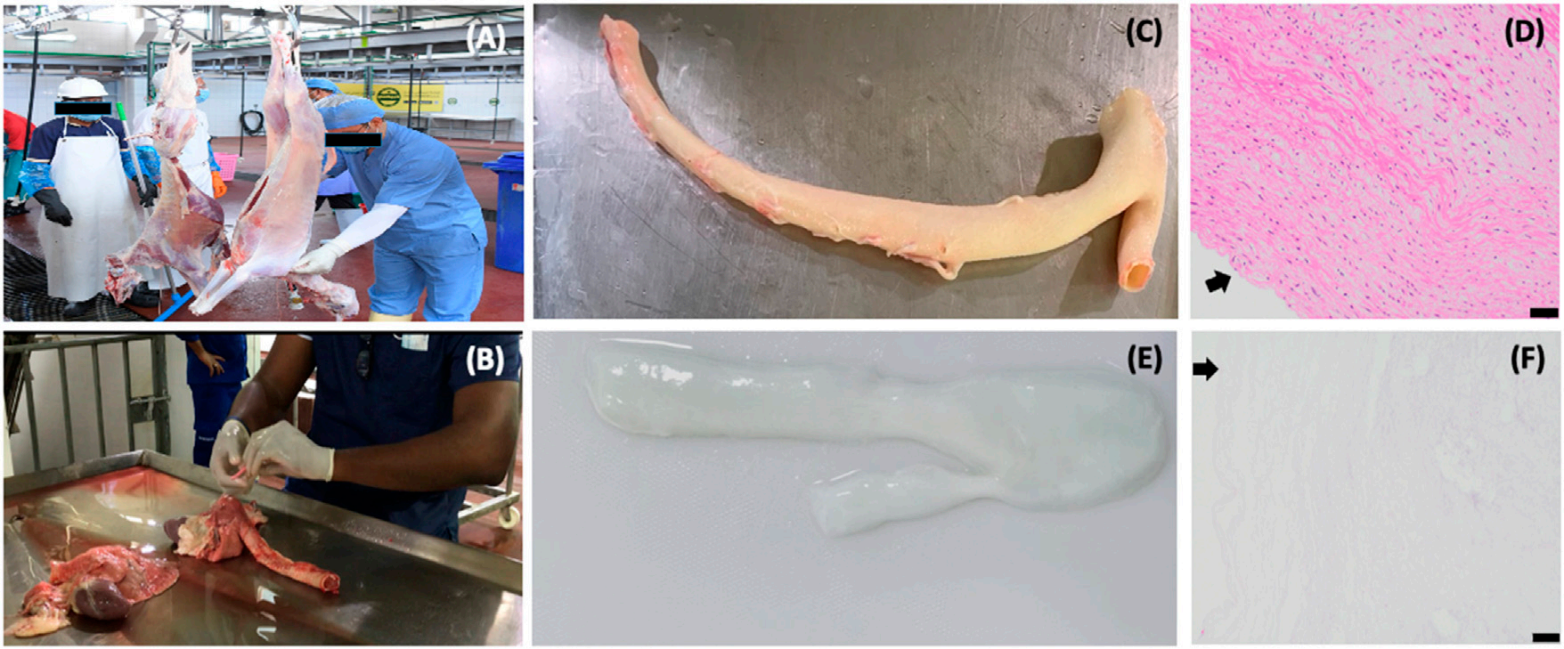
Decellularized vascular scaffolds created from biological waste obtained using native sheep aorta collected at a local slaughterhouse. These images provide an example of the repurposing process, whereby harvested organs are transformed into viable scaffolds for tissue-engineering purposes: **(A)** processing of sheep at the local slaughterhouse for the collection of meat, **(B)** discarded thoracic sections being dissected to obtain the aorta, **(C)** an intact aorta that was dissected and prepared for decellularization directly after it was harvested, **(D)** histologic image of a native aorta shown in image **(C)**, **(E)** aorta after decellularization, and **(F)** histologic image of the decellularized aorta showing the significant variation in cellular components present in the native structure compared to the scaffold **(E)**. Arrows in images **(D)** and **(F)** identify tunica intima and vascular lumen, and scale bars represent 50 µm.

**FIGURE 3:**
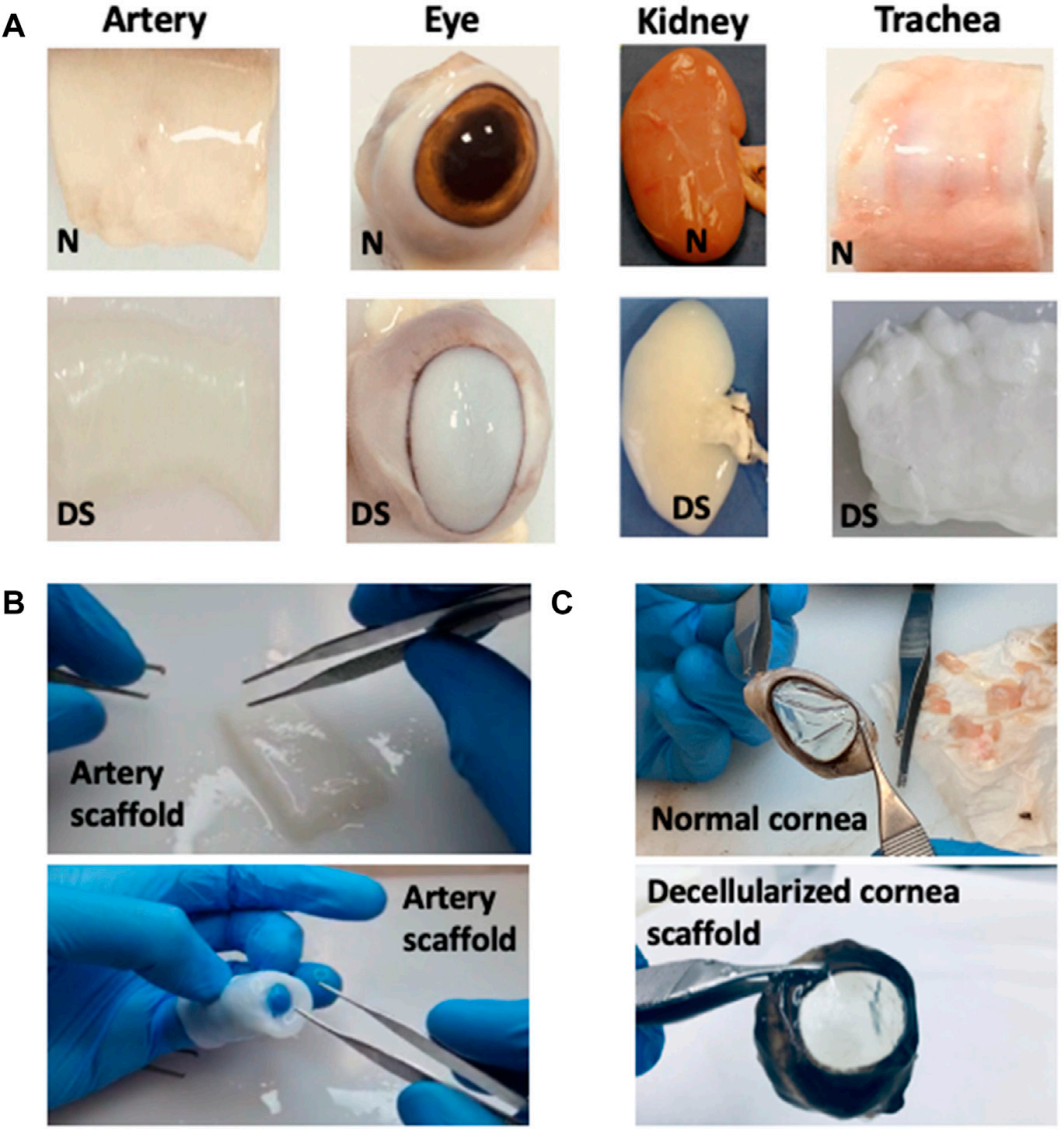
The formation of various tissue and organ scaffolds. These images illustrate the conversion of native tissues/organs into the decellularized **(A)** artery, eye (initially with substantially reduced optical transparency), kidney, and trachea scaffolds, where *N* = native scaffold and DS = decellularized scaffold, **(B)** a decellularized arterial scaffold illustrating signs of retained flexibility and structural integrity, and **(C)** decellularized corneal scaffold opacity reversed to provide a degree of transparency comparable to the native (normal) extracted cornea.

Unfortunately, there are also dangers associated with these approaches, namely the immunological rejection of the organ and endogenous viruses infecting the recipients, and possible strategies to prevent immune responses are discussed later in this manuscript. Consequently, the drawbacks of higher antigenicity and immunogenicity in xenogeneic tissues are mitigated by removing all cellular tissue components *in vitro* using various chemical and physical techniques. Again, this approach is not without challenges since using these acellular or decellularized tissues, such as those presented in Figures 2, 3, requires reseeding or recellularization of the acellular vascular grafts with autologous cells from various sources, including endothelial, smooth muscle, and myofibroblast to yield functional vascular templates (Wilhelmi et al., 2014).

Decellularization is a particularly suitable option compared to other tissue engineering approaches. Vascular template recellularization strategies with autologous cells of various origins were compared to those obtained without prior autologous cell seeding (Khoffi et al., 2014). It was discovered that autologous reseeding seems to protect grafts from degeneration, thrombotic adhesions, and thus early loss of function (Wilhelmi et al., 2014). Remarkably, veins with autologous cells have also been shown to remain patent for up to 2 years after implantation, yet partial vascular narrowing was observed after nine months (Pashneh-Tala et al., 2015). This reduction in patency resulted in the need for a replacement graft. In contrast, grafts with allogeneic or xenogeneic endothelial surface coverage underwent inflammatory and degenerative changes, resulting in total tissue loss. The studies above identify the potential of vascular xenografts obtained from slaughterhouses.

### 2.2 Eyes

Although cadaveric donor corneal grafts are commonly used for transplantation, the mismatch in supply and demand severely limits this option (Choi et al., 2015). Moreover, despite the implementation of tissue quality controls and donor screening, approximately one out of every six full-thickness corneal transplants experience some degree of failure (Achiron et al., 2022). This failure rate could be due to the graft’s rapid opacification, which occurs from the first day to the second or third week after transplantation (Maumenee, 1973; Rowe et al., 2017). Several early failures are also related to technical issues in the surgical method, such as insufficient donor material acquisition, alterations in the anterior chambers, and lens trauma (Alió del Barrio et al., 2021).

In severe corneal blindness that is not conducive to a corneal transplant, keratoprosthetic implants may be utilized as a last resort (Dohlman, 2022). They usually consist of a polymethylmethacrylate optical cylinder that focuses the images on a working retina and a supporting piece that secures the implant to the eye (Avadhanam et al., 2015). However, the main issues stem from the eye’s propensity to extrude biomaterials that are mechanically anchored but not biologically integrated.

A corneal substitute must be biocompatible and have a spatial architecture similar to the native tissue to be optically transparent. It should also be strong enough to withstand manipulation in culture, potential suturing, irrigation, and handling during surgery. Furthermore, it needs to provide a flexible structure to match the shape of the eye and lay flush on the reception surface while facilitating the transfer of nutrients and waste across its structure (Zhou and Caspi, 2010). Since decellularized xenografts do not rely on human donor availability and can potentially reduce the risk of immunogenetic responses, this approach may be an alternative to corneal allografting. Coupling this approach with various cell technologies can help produce patient-specific epithelial and endothelial layers and thus, personalized grafts, (Shaw and Kirk, 2019). Despite this, different species would be preferred to generate viable ocular substitutes based on their anatomical and physiological compatibility so that the intended organ operates efficiently in humans.

Some proposed corneal xenografts (derived from bovine, ovine, and porcine donors) have similar physiological, anatomical, and optical properties to human corneas, and they are relatively easy to obtain in large numbers (Zhou and Caspi, 2010; Sharifi et al., 2019). It should be noted that the risk of cross-species disease transmission, higher rejection rates compared to allograft tissues in similar conditions, and xenograft failure due to immune responses have led to the investigation of human decellularized corneas as an alternative (Zhou and Caspi, 2010; Wilson et al., 2013; Wilson et al., 2016; Polisetti et al., 2021). However, this approach is inherently limited by low donor rates. Interestingly, enhancements in genetic modification technologies and immunosuppressive therapies have significantly improved the survival outcomes of xenografts, while limiting the risk of xenozoonosis in preclinical xenotransplantation models (Ryczek et al., 2021).

Sheep and humans also share vital elements of ocular biology, making them suitable candidates for corneal research. The sheep’s eye has a deep anterior chamber that allows for surgical treatments, and corneal transplants undergo rejection processes that are clinically and histologically identical to those seen in humans. The ovine corneal endothelium is also essentially amitotic *in vivo* (Klebe et al., 2001) and again, comparable to the human corneal endothelium (Al Abdulsalam et al., 2018).

For xenografting in general, non-human primates are the most closely related animals to humans phylogenetically (Estes et al., 2018). However, using these animals as donors introduces drawbacks related to ethical concerns, possible zoonotic illness transmission, high expense and long breeding times, and a variety of uncharacterized genetic alterations. Although the eye is immune-privileged (Zhou and Caspi, 2010; Taylor, 2016), innate, humoral, and cellular immune responses will impact corneal transplant rejection. Such immune responses are seen in corneal xenograft rejection produced by xenogeneic antigens, notably pig antigens (Sharifi et al., 2019; Oh et al., 2022). Immunologic reactivity, graft size, the existence of corneal endothelial cells, and critical differences between the donor and the recipient all affect the survival of a corneal xenograft. Promising trials were performed for lamellar or full-thickness corneal xenotransplantation with or without immunosuppressants to assess the time required for graft survival. It was found that this time varies based on the breed of the donor and recipient and the type of immunosuppressive treatment (Armitage et al., 2019). Aside from immunosuppression, three major approaches were tried to overcome xenogeneic rejection: genetic modification of the source animal, formation of bone marrow chimerism in the recipient, and encapsulation of the xenogeneic cells or tissues (Yoon et al., 2021a).

On the other hand, as we explore the potential of ocular xenografts that can be obtained from slaughterhouses, non-primates like cows, goats, pigs, and sheep offer additional benefits compared to non-human primates that relate to cost, accessibility, and supply (Sharifi et al., 2019). Furthermore, ethical concerns are reduced as these animals are traditionally reared for human consumption, considered the best donor model for xenotransplantation (Hryhorowicz et al., 2017), and at the forefront of evolving genetic modifications to reduce rejection (Ryczek et al., 2021; Allison, 2022).

Porcine corneal xenografts obtained from laboratory animals have exhibited long-term survival in non-human primate experiments, with the longest median survival time exceeding 2.5 years, observed in a full-thickness pig-to-rhesus corneal transplant (Choi et al., 2015; Kim and Hara, 2015). In comparison, xenografts generated from gamma-irradiated decellularized slaughterhouse pig corneal tissues supported human corneal epithelial, stromal, endothelial, and hybrid neuroblastoma cellular growth and differentiation, and *ex vivo* transplantation (Islam et al., 2019). Likewise, *in vivo* studies revealed ideal re-epithelialization, stromal recellularization, and complete transparency post-implantation of abattoir-derived corneal grafts maintained transparency without any signs of rejection during the complete follow-up (Lin et al., 2018).

Luckily, multiple efforts have been devised to address this issue by focusing on preventing pathogen entry into knackeries, as well as advanced detection schemes. For instance, comprehensive e-learning programs on biosecurity can help train and educate various members within agricultural supply chains (Høg et al., 2011). Also, the simple introduction of insect screens (Høg et al., 2011), real-time/microarray PCR systems, miniaturized biosensors, air sampling, chromatographic techniques, and DNA sequencing can improve our monitoring capacity at a lower cost (Søndergaard et al., 2014; Josefsen et al., 2015). The current SARS-CoV-2 pandemic brought new challenges and opportunities in the meat industry and highlighted the emerging role of digitization using data analytics and artificial intelligence (AI) to support the real-time needs (e.g., remote monitoring and management decision tools) of the food industry, smart agriculture, supply chain, and food security *via* robotization and intelligent systems within abattoirs [(Galanakis et al., 2021), (de Medeiros Esper, 2021)]. Lastly, the designation/creation of various sterile regions within the knackeries can also support on-site tissue procurement and transportation to research facilities to support the viability of this approach further.

### 2.3 Kidneys

Transplantation is the optimal treatment for end-stage renal disease, but the global shortage of organ donors limits its clinical application. The bioartificial renal tubule assist device (RAD) was the first synthetic kidney designed to address this scarcity (McKee and Wingert, 2016). Despite advancements over time, these bioartificial kidney systems have several flaws, including problems with blood flow through the apparatus and clogging of pores inside the units (Shaw and Kirk, 2019). Another major drawback of these devices is their extracorporeal nature, which renders them prone to infection and thrombosis. Research is also being conducted on devising improved bioreactor systems, identifying alternative gene and cellular approaches to improve cell and tissue regeneration (Hall et al., 2012; Corridon et al., 2013; Collett et al., 2017; Kolb et al., 2018; Corridon et al., 2021; Cai et al., 2022; Shaya et al., 2022; Corridon, 2023a), and employing AI to enhance detection and diagnostics (Shaw and Kirk, 2019; Pantic et al., 2022a; Pantic et al., 2022b; Corridon, 2022; Corridon et al., 2022; Davidovic et al., 2022; Corridon, 2023b; Pantic et al., 2023b).

As an alternative to externally worn devices, cell therapy techniques were devised in conjunction with whole kidney decellularization to support the repopulation of acellular templates with a patient’s cells (Corridon et al., 2017; Shaw and Kirk, 2019; Corridon, 2021; Pantic et al., 2022a). Eliminating all existing cellular material is one of the fundamental challenges of using complex organ-derived scaffolds (Wijkstrom et al., 2017; Shaw and Kirk, 2019). This step is crucial to ensure that the seeded cells’ subsequent cell proliferation and differentiation are only due to cell-matrix communications and signals between implanted cells, reducing the risk of immune response (Wijkstrom et al., 2017; Shaw and Kirk, 2019).

Kidneys may be harvested from animals and cadavers, and the renal extracellular matrix can be decellularized to potentially provide a platform for subsequent reseeding and production of a semi-functional kidney. The efficacy of this technology in clinical settings is currently limited due to the need to establish universal protocols to support decellularization (Elliott et al., 2012) and renal cellular differentiation needed to repopulate various complex renal compartments (Corridon et al., 2017). While progress has been achieved with bioartificial kidneys and reseeding acellular scaffolds, how well the ECM is conserved between species (Shaw and Kirk, 2019) and the effects of decellularization and sterilization on the underlying structure are still unknown (Łopianiak and Butruk-Raszeja, 2020). As these issues have significant implications for developing whole animal organs for human transplantation, the area still faces significant challenges.

Pig kidneys are very similar to human kidneys in form and relative size, and these animals have been promoted as a potential model to facilitate translational renal investigations, but there are notable physiological discrepancies between the two species. Although numerous xenotransplantation trials in large animal models have been conducted, they have always used immunosuppressive regimes that are more severe than those used in allotransplantation. For instance, the side effects of calcineurin inhibitors are to blame for the loss of kidney allografts due to recipient death while the graft is still functional (Wijkstrom et al., 2017; Shaw and Kirk, 2019). There is also limited evidence that humans can tolerate significantly more stringent immunosuppression than current allotransplantation regimens require (Khazraee et al., 2018a). Investigations in big animal models have revealed that genetic changes can improve the longevity of such xenotransplanted organs (Shaw and Kirk, 2019).

### 2.4 Tracheas

The trachea is a crucial part of the conducting zone within the respiratory system and consists of various tissue types, including cartilage, muscular, vascular, and nervous tissues. This region of the respiratory tract is supplied with arterial blood by branches of inferior thyroid arteries that stem from the thyrocervical trunk. For its proper function, its strength, stiffness, and vascularity are crucial (Khazraee et al., 2018a). The insertion of a vascularized soft tissue flap and the creation of anastomoses between this flap and the trachea adventitia are currently the only appropriate method of tracheal blood supply. However, cartilaginous tissue acts as a barrier to blood vessel ingrowth into the mucosal lining as cartilage is avascular and relies entirely on diffusion. Consequently, the decellularized trachea must be able to support this nutrient/waste transport mechanism.

In tissue engineering, there are various strategies for establishing a blood supply. For instance, the introduction of endothelial cell layer and various growth factors into the bioengineered trachea are likely to speed up neovascularization, as illustrated in tissue-engineered tracheas derived from decellularized porcine jejunum segments containing autologous endothelial cells (Khazraee et al., 2018b). Incorporating angiogenic molecules such as vascular endothelial growth factor, platelet-driven growth factor, and primary fibroblast growth factor into bioengineered structures is another option to promote revascularization (Khazraee et al., 2018a). Overall, it should be noted that further research is needed to devise methods that support homogenous endothelial revascularization patterns that can sustain *in vivo* blood flow.

Initial results from cadaveric allografts prompted the construction of decellularized tracheal scaffolds that possess an ECM and eliminate immunogenic responses in animal models (Wong and Griffiths, 2014). Acellular tracheas maintain the biomechanical qualities of the native organ since the mechanical properties are generally derived from collagen, elastin, glycosaminoglycans, and proteoglycans within the ECM. A cadaveric donor trachea was decellularized in previous studies, with all cellular components, including major histocompatibility complexes (MHCs), successfully removed (Elliott et al., 2012). These findings, combined with reports of preclinical success with similar approaches for heart and lung transplants, show that decellularized scaffold-based technologies could offer a non-immunosuppressive alternative to traditional transplantation (Elliott et al., 2012). Additionally, it was found that airway grafts require early mucosal coverage and mucociliary clearance in patients with compromised bronchial or lung function. Accordingly, research into mechanisms of respiratory mucosa regeneration and the identification of stem or progenitor cells, and migration and differentiation factors are critical for advancing this application (Elliott et al., 2012).

### 2.5 Large intestines and bladder

Existing clinical strategies for intestinal and bladder substitution or reconstruction, which involve resection of damaged sections (Boroni et al., 2022) and the use of autologous segments of gastrointestinal tissue, in order to restore innate function (El-Taji et al., 2015; Chen et al., 2021), respectively, are prone to failure; therefore, novel alternative approaches are needed to address these issues. Advances in bioengineering are helping to engineer suitable scaffolds that can sustain the mechanical forces necessary for autonomic and sensory innervated peristaltic movements and filling and emptying (Serrano-Aroca et al., 2018). Additionally, stem cell technologies may be harnessed to utilize the regenerative cell populations that reside in intestinal (Rees et al., 2020) and bladder tissues (Chan et al., 2017).

The acellular intestinal matrix has been shown to promote constructive remodeling in porcines and rodents *in vivo*, which is a necessary step in tissue maturation and restoration (Han et al., 2009; Nowocin et al., 2016). The ability to use the decellularized ileum as a scaffold in tissue engineering was established *in vivo* in a rat model (Nowocin et al., 2016). The scaffold did not appear immunogenic in that study and acted as an excellent matrix for cellular ingrowth. Up to 8 weeks after implantation, acellular intestinal samples were well tolerated and did not cause significant inflammation or immunological response. The efficiency of the cells and the elimination of their remnants are vital for the host tissue response following the *in vivo* implantation of ECM scaffolds. For instance, using suitable decellularizing agents is crucial. The simultaneous perfusion of multiple enzymes and detergents through freshly harvested ilea successfully removed cellular and nuclear components without significant reductions in the number of functional protein, collagen, and elastin structures throughout the scaffold.

In addition to their potential use as starting materials to create bioartificial scaffolds, harvested intestines and bladders can be used to collect stem cells. The small intestine’s inner epithelial lining is a multifunctional tissue. It must achieve efficient digestion and absorption of food contents released from the stomach while maintaining an effective barrier against potentially harmful microbes and carcinogens in the intestinal lumen (with the help of enzymes released from the liver and pancreas). Daily self-renewal is required throughout life because of the high cell loss rate, fueled by small populations of adult stem cells that live in specific niches (Barker, 2014).

Multipotent mammalian intestinal stem cells reside in the base of the crypts and can be isolated and expanded *in vitro*. Similarly, basal cells in the adult urothelium within the bladder are a type of stem cell capable of renewing and differentiating into intermediate and superficial cells (Lu et al., 2019). Typical properties of urothelial stem cells derived from normal and carcinogenic strains have emerged. Both lineages have been characterized by their remarkable plasticity and reliance on reciprocal interactions with stromal fibroblasts (Hatina and Schulz, 2012). The regenerative capacity of these stem cells makes them excellent candidates for creating various other cell lines, and their carcinoma stem capacity extends their utility in restorative and disease-model research. The directed differentiation of these cells into diverse specialized intestinal cell types offers opportunities for patient-specific applications (Clevers et al., 2019).

### 2.6 Urine and fecal derived stem cells

Stem cells are the body’s raw material, giving rise to all other cells with specialized roles. Bioartificial tissues are created by seeding stem cells or differentiated cells into a natural or artificial biomaterial scaffold shaped in the proper form, then implanting the construct in place of the injured tissue or organ (Lindahl et al., 2015). Available literature discusses the benefits of urine-derived stem cell therapy and tissue engineering applications in regenerating tissues in the genitourinary tract. For example, urine-derived stem cells also secrete multiple growth factors and cytokines and can differentiate into various cells such as podocytes, myocytes, endothelial, and urothelial cells (Bento et al., 2020). This attribute highlights the feasibility of this cell source for use in cell-based therapies to treat tissue abnormalities or disorders in other organ systems (Zhang et al., 2014; Zhou et al., 2021). Likewise, urine- and intestinal-derived stem cells exhibit a phenotype similar to mesenchymal stromal cells and can be converted into induced pluripotent stem cells, which can, in turn, be exploited in the ways mentioned above.

These stem cells can be extracted directly from large intestines and the bladder to develop various bioartificial replacement tissues and organs. Their isolation can be achieved by conventional centrifugation, gradual removal of impurities, and inoculation into culture media to support strong proliferative and multidirectional differentiation capabilities. This non-invasive and simplified procedure represents a novel breakthrough in autologous and donor stem cell research after first being identified by Zhang et al. (2014). Previous studies have detailed the isolation of such stem cells from monkeys, pigs, and rabbits (Zhang et al., 2014), as well as humans (Zhou et al., 2021).

In comparison, to our knowledge, no studies to date have been published on the successful isolation of fecal stem cells. Nevertheless, these lineages are believed to exist (He et al., 2020; Yoon et al., 2021b), and we speculate that comparable endeavors can also focus on the reclamation of stem cells derived from fecal matter that may be of multiple lineages. It is feasible to envision this form of cellular recovery because stem cells are naturally released to heal major intestinal or colon-based pathologies. Specifically, crypt remodeling replenishes intestinal stem cell populations (Bohin et al., 2020) that can, in turn, be translocated after sloughing off individually or in clusters (Williams et al., 2015) from the villus tip to distal intestinal luminal compartments and, ultimately, feces.

The ability to potentially isolate stem cells from these animal waste products provides a novel opportunity to enhance waste valorization by focusing on slaughterhouse wastewater. Efforts can thus be made to acquire stem cells before their active decay or transformation in this effluent. Thus, stem cells derived from urine and fecal matter can be implemented as alternative cell sources and avenues of research to support scaffold development in animals and technology transfer later to human models.

### 2.7 Decellularization techniques

Several techniques over the past 40 years have been investigated to produce decellularized scaffolds from various organs including blood vessels [(Wang et al., 2022a), (Malone et al., 1984)], eyes (Wilson et al., 2013), kidneys (de Haan et al., 2021), and tracheas (Damiano et al., 2021) using physical and chemical treatments. Although decellularization strategies vary widely based on the distinct features of tissues, including structure, components, size, and thickness, they all focus on generating tissue/organ scaffolds composed of natural and hypoimmunogenic ECMs that possess biomechanical properties similar to native tissues (Burk et al., 2014).

Immersive and agitative protocols are commonly used for hollow or less dense structures. This approach has been described for multiple tissue types, including blood vessels, heart valves, skeletal muscle, cartilage, tendons, tracheas, esophagi, dermal compartments, and bladders. The structure to be decellularized is immersed in chambers with decellularizing agents, and agitation is achieved with a magnetic plate, ultrasound source, shaker, or an agitator attached to the end of the chamber. It should be noted that the duration of the protocol of immersive/agitative approaches is a function of the tissue complexity, thickness, density, detergent type, and intensity and duration of applied treatment. Peristaltic pump-driven perfusion regimens can also be applied to enhance further the surface contact and homogeneity of the detergent-tissue interface (Syed et al., 2014).

Whereas, for more complex and vascularized structures, like the kidney, heart, and lung, some form of perfusion treatment is primarily applied in standalone antegrade or retrograde patterns to homogenously distribute the decellularizing agent(s) across the organ. Regardless of the decellularization model, a given treatment’s effectiveness is evaluated by its simultaneous ability to remove cellular remnants and retain essential ECM components efficiently. Effective cellular remnant removal is often evaluated on the remnant DNA contents. At the same time, the intactness of the ECM scaffold is judged by the retention of glycoproteins, proteoglycans, and glycosaminoglycans, as well as structural proteins like collagen, elastin, fibronectin, and laminin (Corridon et al., 2006; Corridon and Wilke 2007a; Corridon and Wilke 2007b; Corridon et al., 2007). These characteristics support the creation of hypoimmunogenic natural ECM-based scaffolds that display biomechanical properties similar to the native tissue and can be used to drive the regeneration process.

## 3 Regulatory approaches needed to support this endeavor

### 3.1 Regulatory framework

The legislative framework for xenotransplantation must also be considered when contemplating using abattoir waste as a starting material and can be derived from conventional approaches. Conventional xenotransplantation approaches, which utilize tissues from laboratory animals, date back over 40 years (Cooper, 2012; Cooper et al., 2015). These efforts have facilitated hundreds of xenotransplants to treat metabolic disorders using pancreatic tissue from pigs, cows, and rabbits. However, the magnitude and effect of such initiatives are still being determined because of poor patient documentation, follow-up, and publication, but they have inadvertently laid the foundation for multinational industrial processes. From a global perspective, xenotransplantation legislation has been developed in North American, European, and Asian nations (Cooper et al., 2017), and there is a growing understanding of the significance of having an internationally unified regulatory framework, which may also be extended to consider slaughterhouse materials as starting agents to develop viable grafts.

Furthermore, slaughterhouse meat and poultry products stem from complex activities requiring good hygiene practices. Regardless of the production scale, such operations must be conducted with veterinary antemortem and post-mortem inspections to minimize the risk of contamination with pathogenic organisms (Authority and Authority, 2019). Pathogens that cause foodborne illnesses can incubate rapidly in these tissues and be detected several weeks after initial contamination. So, it is imperative to ensure the proper handling of offal wastes to reduce the risk of infection that can lie dormant in scaffolds/cellular matter repurposed for bioartificial tissue and organ development.

We argue that analogous practices and training programs must be defined at the governmental level to guarantee that healthy tissues and organs are collected for repurposing. These undertakings will help diminish antigen transmission from infected tissues and fluids that can be used to create xenotransplantable products. The delicate balance between individual and collective rights and globalization tensions necessitates a coordinated international effort to unify international norms in this area. According to the United States Food and Drug Administration (Hayat et al., 2019), xenotransplantation should be reserved for patients with severe or life-threatening conditions for whom there are no acceptable, safe, and effective alternative therapies (Wijkstrom et al., 2017). This reservation is also extended to patients who have the potential to benefit from substantial improvements through clinical treatment and quality of life after the procedure (Wijkstrom et al., 2017). Still, it is hoped that the advances in technologies outlined here can revise and further extend this view.

Most xenotransplantation-based research has been conducted in North America and Europe, with the vast majority of clinical trials performed in the United States (Girani et al., 2019). Nevertheless, recent activity within Asia is supporting the development of this field. Many Asian countries, such as China, Japan, and South Korea, are now conducting extensive studies on this subject (Chen et al., 2003; Guo et al., 2014; Shuji et al., 2017; Wang et al., 2019a; Girani et al., 2019; Kobayashi and Miyagawa, 2019; Pan et al., 2019; Park et al., 2019; Shimoda and Matsumoto, 2019; Kwon et al., 2020a; Kwon et al., 2020b; Liu et al., 2020; Liu and Liu, 2021; Sung et al., 2021; Huang and Foundation, 2022). Sociocultural factors are correlated to significantly lower organ donation rates in this region compared to Western countries. With many Asians’ shared reluctance towards being separated from their organs after death (Li et al., 2019), xenotransplantation is now becoming a viable option (Ekser et al., 2015). Successful preclinical pig‐to‐nonhuman efforts have laid a niche foundation for upcoming primate corneal transplants in this region. It is hoped that such efforts can support further development of other organ-based xenogeneic activities.

Since pigs have also taken the role of non-human primates as possible donors, clinical trials involving transplanting non-human primates’ organs to humans are no longer permitted (Girani et al., 2019). In comparison, the guidelines provided by the European Medicines Agency (Rostami et al., 2022) and FDA, as opposed to rules and directives, indicate the regulatory authorities’ current thinking on a particular subject and are therefore open to debate and flexible interpretation with different approaches for particular product development. Thus, it is unclear how this will be accomplished (Schuurman, 2015).

Within the past two decades, the European Union (EU) has supported the use of xenogeneic materials by classifying xenogeneic cell therapy and materials as advanced therapy medicinal products. The EU has also adopted an associated regulatory framework in 2007 associated with EMA guidelines that outlines general principles for the development, authorization, and pharmacovigilance of xenogeneic cell-based medicinal products. Overall, these documents stress the importance of quality and manufacturing; nevertheless, according to the European Parliamentary Research Service (EPRS), no significant and recent legislative developments related to xenotransplantation have been implemented by the EU (Quaglio et al., 2022). While the regulation mentioned above defined a significant and essential step towards a unified legal and ethical EU framework on xenotransplantation, a more comprehensive regulatory approach is needed for this promising practice to reach its full potential (Quaglio et al., 2022).

### 3.2 Biosecurity and ethics

**FIGURE 4:**
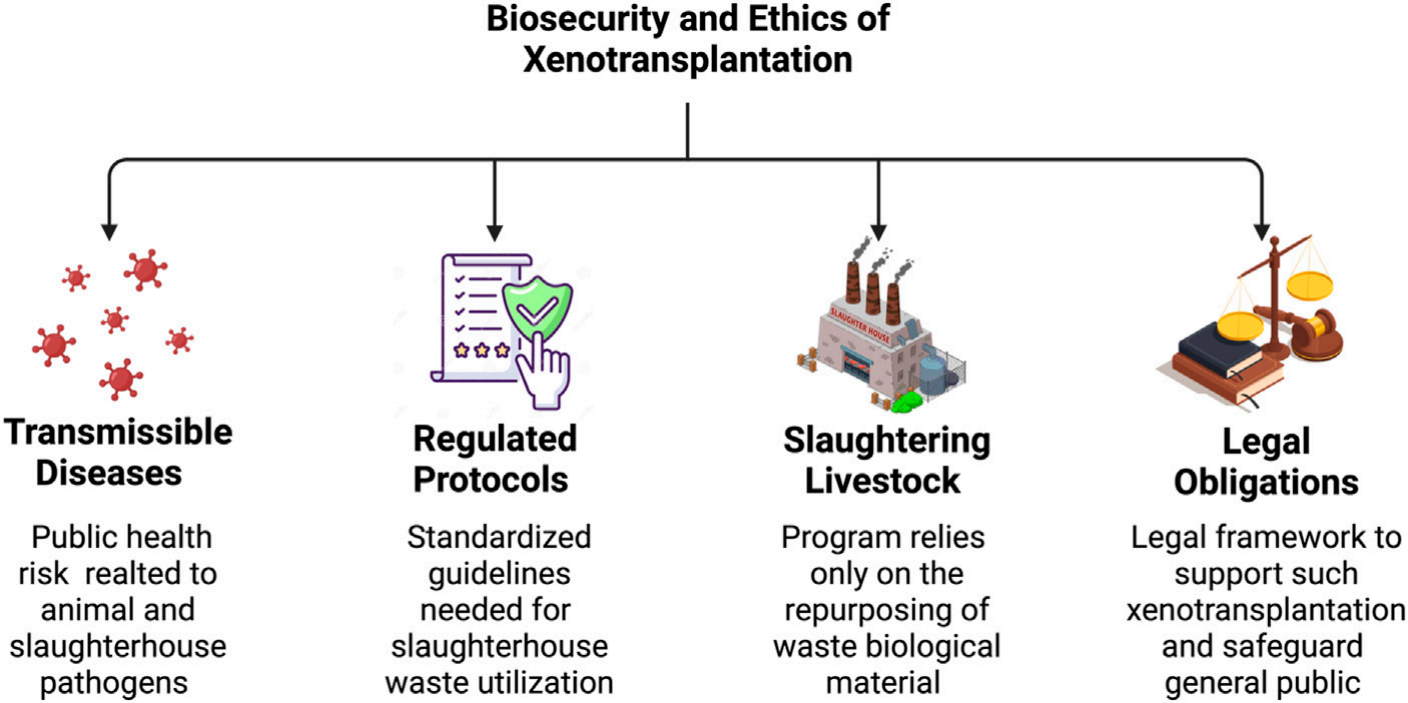
Biosecurity and ethical considerations for utilizing slaughterhouse waste in xenotransplantation. The figure above provides a brief overview of the ethical aspects needed to support the development of xenografts using slaughterhouse waste, including the risk of transmissible diseases, regulated protocols needed to be followed, the humane and sustainable slaughtering of livestock, and legal obligations.

After xenotransplantation, the risk of exposure to endogenous viruses will emerge. Such exposure may affect transplant recipients and the general population since some diseases can spread throughout a community. These events pose a public health risk, as shown in Figure 4. Concerns for the transplant recipient’s safety and public health severely limit the privacy of such recipients, as they must be checked for viruses that are deadly to them and others for extended periods. Therefore, safety and ethical conflicts can arise while balancing patient and societal benefits (Melo et al., 2001).

Slaughterhouses are potential reservoirs of bacterial, parasitic, prion, and viral pathogens (Franke-Whittle and Insam, 2013). These pathogens can infect both animals and humans. As a result, the proper handling of animal carcasses has always been and continues to be a concern for the food and waste management industries. Thus, the use of this form of waste has several limitations compared to the conventional acquisition of tissues/organs from laboratory animals. Specifically, slaughterhouse environments are incompatible with the pathogen-free environments required for biomedical applications. As a result, this issue needs to be addressed in a manner that would not adversely influence the price of meat and food sustainability.

Recently, the identification of slaughterhouse waste as potential energy sources that can reduce the reliance on petroleum-based fuels has prompted the need to re-examine biosecurity measures for support initiatives beyond disposal. In this case, biosecurity refers to measures aimed at minimizing the risk to prevent the introduction or spread of harmful organisms to various individuals that come into contact with these tissues (Vinneras et al., 2012), particularly researchers, clinicians, and recipients, and in turn, the wider community.

Although modern pathogen-free facilities and thorough testing have alleviated particular concerns for transplantation, issues related to exogenous retroviruses incorporated into the xenograft’s genome remain (Wijkstrom et al., 2017). Evidence has been presented on how such retrovirions can be eradicated using gene-editing techniques and that these microbes can be managed with currently available antiretrovirals. Thus, the risk of infections arising from xenotransplantation may be comparable to allotransplantation. Nevertheless, alternatives to traditional approaches that yield high levels of pathogen inactivation, like anaerobic digestion, alkaline hydrolysis, rendering, incineration, and burning, must be devised to ensure the overall minimization of such risks.

Another component to developing a process to create bioartificial tissues and organs from slaughterhouse waste would be establishing methods to ensure the proper oversight of this waste through appropriate legislation (Tritt and Schuchardt, 1992). Based on the fact that these animals will be slaughtered regardless of repurposing effects and scientific involvement, guidelines applicable to such research may include:

• careful consideration of species and numbers requested;
• qualifications of personnel;
• licensing and/or certification; and
• proper handling of tissues to minimize the risk of pathogen transmission and tissue damage.

When developing a protocol for using abattoir waste as a starting material for xenotransplantation, appropriate guidelines can be given by an Institutional Animal Care and Use Committee (IACUC) and an Institutional Review Board (IRB) (Triller and Bobinski, 2004).

Animal research should be performed in the most humane manner possible with the minimum number of animals required to obtain valid results. Even though these procedures do not apply to slaughterhouse animals, a new framework that focuses on obtaining tissues and organs from animals that are not explicitly slaughtered for the activity should be implemented. The humane treatment of animals in research also encompasses how the animals are housed. Although the environments in which animals reared solely for organ transplantation are housed are far preferable to agricultural circumstances regarding animal health, they are also inadequate in satisfying the animals’ biological and psychological needs (Rollin, 2020). Considering that the animals used in this research will be acquired from slaughterhouses, the humane butchering of these animals should also be sought after.

Xenotransplantation and slaughterhouse waste collection centers should be legally obligated to observe public health rules that safeguard the general public from xenozoonosis (Triller and Bobinski, 2004). National public health bodies, such as the FDA and the Public Health Service (PHS) (Elliott et al., 2012), specify standards of conduct in xenotransplantation, and their regulations are modified in response to scientific discoveries (Lindahl et al., 2015). One of the essential variables to consider when using this aspect in a case of negligence is how well these centers followed the existing standards (Triller and Bobinski, 2004).

The main issue for regulatory authorities is determining the precise definitions and suitable degrees of standards (Sharifi et al., 2019). Even though highly unlikely in highly regulated practices, a porcine microbe may infect the patient and then be passed on to close contacts, such as family or hospital staff, and spread into the community (Wijkstrom et al., 2017). As a result, xenotransplantation clinics can be held liable in the event of violations. Establishing specific insurance schemes and legislature may be sufficient to preclude unsubstantiated claims and might also be used to limit the damages incurred through negligence (Triller and Bobinski, 2004). Following FDA and PHS guidelines, being transparent and truthful to the public, and developing an insurance system would be among the legal defenses that can be utilized (Triller and Bobinski, 2004; Wijkstrom et al., 2017). This approach may promote positive public perception and help develop the xenotransplantation industry.

### 3.3 Slaughterhouse wastewater

The treatment and disposal of slaughterhouse wastewater are both an economic and a public health necessity. The United States Environmental Protection Agency (US EPA) has classified slaughterhouse wastewater as some of the most environmentally harmful industrial waste because improper disposal can cause river deoxygenation and groundwater pollution (Ciro and Mehrab, 2017). As a result, slaughterhouse wastewater requires extensive treatment before being safely and sustainably released into the environment.

Key quality indicators used to assess wastewater strength or quality include biochemical oxygen demand (BOD), total suspended solids (TSS), and fat/oil/grease (FOG) values. Anaerobic treatment is the preferred biological form of management because it effectively handles high-strength wastewater (i.e., wastewater with elevated BOD, TSS, and FOG levels) like slaughterhouse wastewater while requiring less complicated equipment. Although anaerobic treatment is effective, anaerobically treated effluents require posttreatment to meet discharge limitations where complete stabilization of organic matter is not attainable with anaerobic treatment alone (Ciro and Mehrab, 2017).

In comparison, aerobic techniques are commonly used posttreatment for anaerobic effluents and nutrient removal because oxygen requirements and treatment times are directly related to an increase in wastewater strength. Advanced oxidation processes could be applied to improve the biodegradability of wastewater and inactivate harmful bacteria and viruses that remain after biological wastewater treatment. These processes are secondary or tertiary treatment solutions for slaughterhouse wastewater, with excellent overall treatment efficiency for water reuse. This form of treatment includes gamma radiation, ozonation, ultrasonic technology, UV/H2O2, UV/O3, and photocatalysis, resulting in the oxidation and destruction of organic materials (Ciro and Mehrab, 2017).

Accordingly, it is necessary to investigate the abovementioned aspects to support the collection of fecal- and urine-derived stem cells, which possess characteristics similar to mesenchymal stromal cells (MSCs) (Pavathuparambil Abdul Manaph et al., 2018; Mei et al., 2020; Rees et al., 2020; Zhou et al., 2021), including their doubling time and immunophenotype. Specifically, it would be helpful to directly collect this already complex fluid and ensure its complexity is not further increased by allowing it to combine with other forms of wastewater. This approach can potentially provide opportunities to maximize stem cell reclamation for repurposing efforts. Moreover, such efforts would support the development of this technology which can then be translated to humans *via* patient-specific reclamation techniques. It may also be advantageous to initially conduct this form of research in animal models based on current varied regulatory policies revolving around using stem cells that regulate research, sources, and applications across the globe (Al-Tabba’ et al., 2020; Alden, 2007; Wright et al., 2021; Jose et al., 2020; Kimmelman et al., 2016; Dhar and Hsi-En Ho, 2009; Lovell-Badge et al., 2021).

### 3.4 Preserving harvested organs

Regardless of the large volume of waste produced by slaughterhouses annually, the quality and usability of this waste come into question. Inspections in abattoirs, in general, give valuable information about meat quality and do not require many resources. However, there are limitations because disease and condition identification based on gross pathology has low sensitivity and specificity. Therefore, some conditions are likely to be overlooked during routine inspections (Tembo and Nonga, 2015).

Additionally, devising a system by which organs can be harvested successfully is crucial. If the organs are extracted improperly, they will be rendered useless. Improper organ extraction can include accidentally cutting tissues, collecting incorrect organs, and dissecting incomplete organs, for example, getting a kidney without an intact renal artery and renal venous segments. In order to mitigate these risks, trained staff or volunteers should be present at the time of removal. Collaborative Institutional Training Initiative (CITI) modules can be developed so that abattoir workers can be guided accurately. Before the extraction procedure, researchers will learn tissue handling and transportation procedures through mandatory animal use training modules and laboratory sample handling before giving them access to the slaughterhouse. Moreover, all procedures will be overseen by the Principal Investigator. Additionally, researchers will be educated on the necessity of following slaughterhouse policies and procedures, and if they do not, they will be removed from the sample collection process.

Utilizing solid organ waste can also be challenging as tissues quickly degrade without a blood supply. The susceptibility of different organs to degradation varies. Therefore, necessary measures must be implemented to preserve organs after animals are slaughtered. After harvesting, organs can be placed on ice to slow their degradation, and then transferred to the research facility for processing, as shown in Figure 1.

Before decellularization, *ex vivo* perfusion can be used as an alternative method of preserving harvested organs instead of cooling, as shown in Figure 1. This method is advantageous because it reduces cold ischemia times, provides an opportunity for quality assessment, and decreases organ degradation (Tembo and Nonga, 2015). The development of *ex vivo* organ perfusion systems has been extensively researched using multiorgan procurement techniques, which can maintain organ viability comparable to levels observed in organs donated from circulatory death models (Dengu et al., 2022). Slaughterhouse porcine organs, in particular, have been used in several *ex vivo* perfusion investigations.

Efforts are also being made to develop portable perfusion systems that can be adapted from clinical systems used in human organ transplants to support bioartificial organ sustainability. For example, Tolstykh et al. (2010) reported a preservation technique that appeared to provide an effective preservation environment for kidneys in conjunction with being smaller, lighter, and more straightforward, allowing for possible portability. Koshida et al. (2006) created a novel perfusion system that, compared to other coronary perfusion systems, required simpler apparatus and could allow active arterial blood perfusion into the coronary artery.

### 3.5 Possible strategies to prevent immune responses

According to Gilpin and Yang, (2017) two primary components identified to elicit immunogenic responses post-decellularization are remnant genetic materials, such as DNA and RNA, and antigens. The immunological barrier is arguably the strongest obstacle to the clinical application of these materials. If this issue is not sufficiently reduced, the resulting *in vivo* rejection will lead to functional failure of a graft and the need for immediate replacement or removal. Nevertheless, it is still essential to recognize that decellularization alone has been demonstrated to prevent immune responses, as immunological concerns have been a halting point for the widespread use of scaffolds generated from this technology in clinical applications (Nouri Barkestani et al., 2021).

Regarding genetic remnants, Crapo et al. (2011) defined a threshold for effective decellularization. This threshold, which focuses on DNA-based nucleic components, outlines the following criteria that have been commonly accepted to avoid adverse cell and host responses: less than 50 ng dsDNA per mg ECM dry weight, less than 200 bp DNA fragment length, or absence of visible nuclear material in tissue sections stained with 4’,6-diamidino-2-phenylindole (DAPI) or H&E (Crapo et al., 2011).

Such focus on estimating residual DNA concentrations from scaffolds is justified because endogenous components of dead cells activate the immune system through extracellular and intracellular pathways. Specifically, within the post-transplantation environment, circulating phagocytic cells that enter scaffolds will attempt to engulf these remnants, and such materials can be transferred to lysosomes for degradation and recycling. However, inefficient engulfment of remnant DNA components activates the immune responses, which can ultimately result in conditions like systemic lupus erythematosus (Nagata et al., 2010). This condition is the most common form of lupus, characterized by autoimmune activity that results in widespread inflammation and structural damage in the transplanted graft and nearby native regions. Likewise, severe anemia and chronic arthritis can also result in improper DNA and RNA degradation *via* the activation of innate and adaptive immune responses (Nagata et al., 2010; Uehata and Takeuchi, 2020).

Mechanistically, it is likely that unengulfed remnants can induce a form of secondary necrosis (Silva, 2010), which activates the immune system to produce autoantibodies, which can, unfortunately, be amplified by cytokines produced by macrophages in response to the necrotic event (Shao and Cohen, 2011). In addition to RNA and DNA fragments, residual proteins within the scaffold can induce similar dangers, especially with respect to the xenogeneic decellularized ECM. These individual components of the scaffold can elicit a specific cytokine response by host cells after implantation (McInnes et al., 2022).

As a result, it is necessary to devise ways to address these concerns that inherently exist beyond adhering to the decellularization threshold. Despite the availability of highly effective immunosuppression regimens, there is still substantial failure associated with allografting that can be attributed to the diversity of individuals within and between species (Platt et al., 2018). Such diversity creates incompatibilities not amenable to the broad-spectrum immunosuppression (Platt et al., 2018) often used to address autoimmune disorders (Blair and Duggan, 2018). Therefore, it would be useful to generate approaches akin to the treatments for the conditions mentioned above and patient-specific approaches to fine-tuning the management of the immune response to a transplant (Popoola et al., 2014). Additionally, inhibition of b-cell and plasma cell activation *via* plasmapheresis and monoclonal antibody medications may produce graft desensitization protocols that combat the humoral responses, inhibit complement activation, and modulate cell-mediated immunity (Webber et al., 2011) to ultimately improve the clinical utility of decellularized tissues/organs derived from slaughterhouse waste.

Immunogenicity and antigenicity play crucial roles in the host’s immune response to biomaterials generated using decellularization. As previously mentioned, antigenicity, which is the ability of an antigen to induce an immunological response when encountered by the human body (Zhang et al., 2015), is another source of concern. Antigenic determinants, or epitopes, can trigger innate and adaptive immune responses that sensitize the body to foreign antigens present within the transplanted scaffold (Chakraborty et al., 2014). While research has shown that detergent-based decellularization approaches are effective in removing ECM antigens (Wu et al., 2022), other approaches have been devised to enhance the antigen removal process post-decellularization.

The most common antigen removal methodology currently used for clinical applications relies on chemically cross-linking the proteins within xenogeneic acellular tissues with sterilants like glutaraldehyde and genipin (Cissell et al., 2014; Heng et al., 2021). Surface coating approaches are intended to make the xenogeneic proteins unrecognizable to a human patient’s immune system and reduce immunogenicity (Cissell et al., 2014; Wang et al., 2016a) while supporting recellularization (Yao et al., 2020; Heng et al., 2021), as well as the structural and rheological properties of biomaterials (Wang et al., 2019b).

Nevertheless, there are individual and inherent limitations to the treatments geared toward addressing immunological barriers associated with slaughterhouse waste. As previously mentioned, the decellularization process is incapable of generating scaffolds entirely devoid of antigens, and there is also evidence that coating treatments do not entirely mask antigens (Cissell et al., 2014; Heng et al., 2021). Therefore, combining an initial antigenic removal process like detergent-based treatments with chemical cross-linking can improve the longevity of xenogeneic grafts.

Recalling the benefits of inducing genetic modifications, it is also helpful to incorporate this approach to overcome the current immunological barrier. For instance, modifying the class I swine major histocompatibility complex is possible by disabling the beta-2-microglobulin activity to offset immune responses using transcription activator-like effector nucleases (TALEN)-derived genome editing technologies (Wang et al., 2016b). Within this context, such technologies will be more useful during the recellularization process to modify transplanted cells to withstand the transplantation environment better and utilize synthetic biology for the design of bespoke ECMs (Phillips and Tang, 2008; Grant et al., 2018). Ultimately, advances that will support the use of slaughterhouse waste for regenerative medicine purposes will be derived from optimized combinative strategies to prevent immune responses.

## 4 Conclusion and future perspectives

Slaughterhouse waste is an abundant resource that can be utilized as a starting material in xenotransplantation. Decellularizing xenogeneic tissue is an investigated method of mitigating the shortage of transplantable blood vessels, eyes, kidneys, and tracheas. Additionally, urine and fecal-derived stem cells acquired from the large intestines, bladder, and slaughterhouse wastewater can aid in the creation of functional bioartificial structures. However, factors such as mimicking native tissue architecture, biocompatibility, and vascularization hinder these structures’ transition and effective utilization in clinical settings. Procurement and preservation of abattoir offal and the regulatory framework to support their utilization are more factors that must be considered to foster xenotransplantation.

If not disposed of properly, animal-derived waste could seriously threaten the environment and its inhabitants. In an attempt to address this, prior studies have outlined the utility of slaughterhouse waste for biodiesel (Chakraborty et al., 2014) and biogas (Ware and Power, 2016) production, fertilizer-based phosphorus sustainability (Darch et al., 2019) and crop biofortification (Ragályi and Kádár, 2012). Recent advancements in biotechnological and biomedical interventions, such as those presented by Tarafdar et al. (2021) have outlined the transformation of waste substrates from poultry, cattle, sheep, goat, and pig into diversified biomaterials. An example of waste valorization outlines the practical fabrication of biopolymers, composites, heart valves, collagen, scaffolds, pigments, and lipids, among other industrially important biomaterials.

Concomitantly, the discovery and application of urine-derived stem cells presented by Bento et al. (2020), Zhang et al. (2014), and Zhou et al. (2021) demonstrate their capacity for self-renewal and multidirectional differentiation. These characteristics form the basis for unique and versatile progenitor sources for cell-based therapy, tissue engineering, and regenerative medicine. In so doing, we propose the amalgamation of slaughterhouse waste-derived tissues, organs, and stem cells and IACUC, IRB, and additional regulatory framework to devise a novel regenerative medicine platform that can ultimately be translated to human studies.

Based on the views outlined in this manuscript, it is clear that substantial efforts are required to drive this technology forward. Nevertheless, the current state-of-the-art has progressed immensely, and the techniques described herein to repurpose these biological constructs routinely to natural scaffolds are available. Specifically, advances in stem cell technologies provide ways to support differentiation into various lineages combined with advanced gene-editing tools to improve the viability of xenogeneic grafts for long-term transplantation. We envision that such approaches can be mirrored using tissue engineering systems dedicated to bioartificial platforms from the cellular to the tissue, and eventually, the organ level.

In summary, the growing shortage of transplantable organs and tissues has prompted the need for alternative solutions to address various pathological conditions worldwide. This need is further highlighted by the high rates of morbidity coupled with social, psychological, and economic burdens associated with current techniques used to generate autologous grafts. Although advances in tissue engineering technology have promised to produce viable replacement segments and organs with properties comparable to native structures, the gap between theory and application is still significant. Alternatives like repurposing slaughterhouse waste provide synchronized opportunities to foster environmental change and waste valorization that may one day help balance donor organ supply and demand.

## Data Availability

The original contributions presented in the study are included in the article/Supplementary Material, further inquiries can be directed to the corresponding author.
